# Detection of Sub-pT Field of Magnetic Responses in Metals and Magnetic Materials by Highly Sensitive Magnetoresistive Sensors

**DOI:** 10.3390/s25030776

**Published:** 2025-01-27

**Authors:** Hyuna Ahn, Ayana Tanaka, Yuta Kono, Suko Bagus Trisnanto, Tamon Kasajima, Tomohiko Shibuya, Yasushi Takemura

**Affiliations:** 1 Department of Electrical and Computer Engineering, Yokohama National University, Yokohama 240-8501, Japan; 2 Advanced Products Development Center, Technology and Intellectual Property HQ, TDK Corporation, Tokyo 103-6128, Japan

**Keywords:** non-destructive testing, magnetic particle imaging, magnetoresistive sensor, eddy current, magnetic nanoparticles, AC magnetic susceptibility, magnetization relaxation

## Abstract

We developed a measurement system capable of detecting magnetic responses in various material samples. The system utilizes an excitation coil to apply an alternating magnetic field within the frequency range of 1–10 kHz. The magnetic field generated in the samples was detected using a highly sensitive magnetoresistive sensor. The system demonstrated a detection lower limit in the sub-pT range for magnetic fields arising from magnetic responses such as eddy currents and magnetization changes. The frequency dependence of the detected signal intensities correlated well with the physical mechanisms underlying the magnetic responses. Notably, the distance between the excitation coil and the magnetic sensor was maintained at 300 mm. These results, which demonstrate the detection of a sub-pT magnetic field using a highly sensitive magnetic sensor, have not been previously reported and provide valuable insights for advancing practical applications in non-destructive testing and clinical diagnostic imaging.

## 1. Introduction

Non-destructive testing (NDT) is widely utilized in various applications, including production processes, quality control, and deterioration diagnosis. Major NDT techniques include liquid penetrant testing, ultrasonic testing, radiography, and eddy current methods [1,2]. In this study, we present a material detection method using a highly sensitive magnetic sensor. This method also shows potential for applications in magnetic particle imaging (MPI) [3], a promising medical imaging technology. MPI employs magnetic nanoparticles (MNPs) as tracers, which accumulate in diagnostic targets such as tumors. Unlike positron emission tomography, which uses radioisotopes as tracers, MPI is expected to emerge as a tracer-based diagnostic technology without the use of radioactive materials.

In both NDT and MPI, the ability to detect materials with high sensitivity is essential. Additionally, the capability to measure large objects remains a critical challenge for these applications. This study aims to determine the lower detection limit of the magnetic field for identifying conductive/non-conductive and magnetic/non-magnetic materials using a measurement system designed for large-sized objects. To investigate this limit, we conducted measurements of magnetic fields generated by objects using a highly sensitive magnetoresistive (MR) sensor. The system successfully detected magnetic fields in the sub-pT range, reflecting the electrical conductivity and magnetic properties of the test materials, as well as their dependence on the applied magnetic field intensity and frequency. The results, derived from experiments conducted in a low-field range below 1 pT, comprehensively reveal the material and field-dependent characteristics of these magnetic responses. These findings, which have not been previously reported, represent a significant advancement in this field and offer valuable insights for developing practical technologies in both NDT and MPI.

For MPI, the development of clinically applicable systems remains an active area of research. While preclinical MPI scanners for small animals are commercially available [4,5], human-head-sized and body-sized MPI scanners are still under development [6,7,8,9]. To prevent vascular obstructions caused by coagulated MNPs, superparamagnetic iron oxide nanoparticles are used as tracers in clinical applications. Small amounts of iron oxide are considered non-toxic to the human body. However, as the size of MPI scanners increases, generating sufficiently intense magnetic fields and detecting weak magnetic fields from the magnetization response of iron oxide nanoparticles becomes increasingly difficult [10]. Therefore, the detection of MNPs using a human-sized measurement system remains an important and challenging research focus.

In this study, we investigated the magnetic fields generated by the magnetization response of MNPs using a measurement system comprising an excitation coil and an MR sensor separated by a distance of 300 mm. The frequency dependence of the detected signal intensity, attributed to the Brownian relaxation of the MNPs, was successfully observed. These results, obtained using a highly sensitive MR sensor, represent a significant step toward the advancement of human-sized MPI systems.

## 2. Magnetic Response to the Applied Magnetic Field

Eddy currents are generated when an alternating magnetic field is applied to a conductor. According to Faraday’s law, loops of electric current are induced within the material in response to changes in the magnetic field. The magnitude of the eddy current is described by Equation (1), where *I*, *R*, and *Φ* represent the eddy current, the resistance of the material, and the magnetic flux, respectively:

(1) $$I=\frac{1}{R}\frac{d\Phi }{dt}$$

The magnetization change in response to the applied magnetic field can be detected using either a magnetic sensor or a detection coil. the magnetization direction tends to align with the applied magnetic field to minimize Zeeman energy. For magnetic nanoparticles (MNPs) composed of single-domain particles smaller than 30 nm, which exhibit superparamagnetic behavior, two primary mechanisms of magnetic relaxation are typically observed: Brownian relaxation and Néel relaxation [11,12,13]. Brownian relaxation arises from the physical rotation of the particle itself, which occurs in fluid samples. Conversely, Néel relaxation involves the rotation of the magnetic moment within the particle. The relaxation times for these magnetization processes, *τ*_B_ and *τ*_N_, are described by the respective equations provided below:

(2) $$\tau_{B}=\frac{3\eta V_{h}}{k_{B}T}$$

(3) $$\tau_{N}=\tau_{0}\exp\left(\frac{KV_{c}}{k_{B}T}\right)$$

Here, *η* represents the viscosity of the MNP solution, whereas *V*_h_ and *V*_c_ denote the hydrodynamic and core volumes of the MNP, respectively. *τ*_0_ is the attempt time. *k*_B_*T* represents the thermal energy, and *K* is the magnetic anisotropy constant. The effective relaxation time, *τ*_eff_, can be defined by combining these two relaxation times:

(4) $$\frac{1}{\tau_{eff}}=\frac{1}{\tau_{B}}+\frac{1}{\tau_{N}}$$

For the MNPs of 30 nm or smaller used in this work, *τ*_B_ and *τ*_N_ are on the orders of 1 ms (corresponding to a frequency of 1 kHz) and 1–10 ns (corresponding to a frequency range of 0.1–1 GHz), respectively.

The magnetic properties of the magnetization change induced by an applied alternating magnetic field are also characterized by the AC susceptibility, expressed as a complex number. The real and imaginary components of susceptibility are defined as follows [14]:

(5) $$\chi^{\prime }=\frac{\chi_{0}}{1+{\left(2\pi f\tau_{eff}\right)}^{2}}$$

(6) $$\chi^{\prime \prime }=\frac{\chi_{0}\cdot 2\pi f\tau_{eff}}{1+{\left(2\pi f\tau_{eff}\right)}^{2}}$$

Here, *χ*_0_, *χ*′, and *χ*″ represent the static, real, and imaginary parts of susceptibility, respectively. From Equation (6), *χ*″ is expected to reach its maximum when $2\pi f\tau_{eff}=1$.

## 3. Experiments

### 3.1. Measurement Samples

We measured four types of materials in our experiments: aluminum, stainless steel, ferrite, and MNPs. Eddy currents were induced in the metallic samples, specifically aluminum and stainless steel. For this study, we used a ferritic stainless-steel sample, SUS 430, which contains 18% chromium. The ferrite sample was composed of MnZn ferrite, a material typically used as a coil core and in other applications. Notably, the ferrite sample is not electrically conductive.

The aluminum and stainless-steel samples were plates with dimensions of 10 mm × 10 mm and a thickness of 0.5 mm. Additionally, we prepared an aluminum foil sample measuring 15 mm × 15 mm with a thickness of 12 μm. The ferrite sample was cylindrical, with a length of 30 mm and diameter of 4 mm. However, the sample sizes were not essential parameters in this work. Instead, the skin depth and thickness of the conductive samples were critical factors, as discussed in Section 3.2 and Section 4.2. We measured the intensity of the magnetic field generated and propagated from the samples and experimentally determined the detection limit of the field.

The MNP sample was in a liquid phase and consisted of iron oxide nanoparticles dispersed in water. We used Resovist^®^, a commercially available product (FUJI FILM RI Pharma Corp., Tokyo, Japan) clinically employed as a contrast agent for magnetic resonance imaging [15,16]. Resovist^®^ is a hydrophilic colloidal solution containing γ-Fe_2_O_3_/Fe_3_O_4_ nanoparticles with diameters of 5–30 nm coated with carboxy dextran. The original Resovist^®^ solution had an iron density of 26 mg-Fe/mL. To prepare samples with iron contents ranging from 0.028 to 16.8 mg-Fe, we diluted or concentrated Resovist^®^. These samples exhibited varying particle densities, which were expected to result in different degrees of magnetic interaction between particles, leading to variations in their magnetic response.

### 3.2. Measurement Method

**Figure 1:**
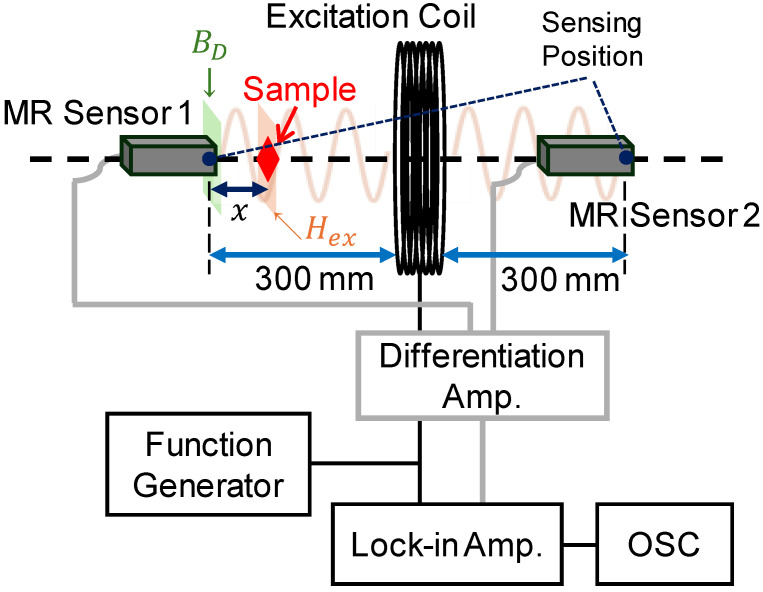
Schematic of the measurement system. The distance between the excitation coil and the sensing position of each MR sensor was 300 mm. An alternating magnetic field with an intensity of *H*_ex_ was applied to the measured sample. The MR sensor (left) detects the magnetic flux density, *B*_d_, generated in the sample. The distance between the sample and the sensing position of the MR sensor was *x* = 20 mm for the aluminum, stainless-steel, and ferrite samples and *x* = 5 mm for the magnetic nanoparticle samples.

Figure 1 illustrates the measurement system used in this study. An alternating magnetic field was applied to the samples using an excitation coil. The diameter, width, and number of turns of the coil were 250 mm, 16 mm, and 320 turns, respectively. The amplitude and frequency of the magnetic field applied to the samples were *H*_ex_ = 0.2–120 nT/μ_0_ (0.16–95.5 mA/m) and *f* = 1–10 kHz, respectively. This field amplitude was determined by measuring the magnetic field using an MR sensor placed at the sample position. The sensing area of the sensor is less than 10 × 10 mm^2^. As the distance between the excitation coil and the sample was 280 or 295 mm, the spatial distribution of the magnetic field generated by the excitation coil was not critical. We define the magnetic field amplitude at the position of the sample. Details of the MR sensor and sample positioning are described in the next paragraph. The magnetic field intensity and frequency were adjusted by controlling a function generator (NF Corp., Yokohama, Japan, WF1947). In this paper, the magnetic field intensity and magnetic flux density are reported using their peak amplitudes. For the measurement of the MNP sample, the applied magnetic field intensity was 1.3 μT/μ_0._ The skin depth of the applied magnetic field at 10 kHz in aluminum was 0.8 mm, allowing the field to penetrate the aluminum and stainless-steel samples, both of which were 0.5 mm thick. However, a skin effect was observed at *f* > 5 kHz in the aluminum sample, as discussed in Section 4.2. The applied magnetic field also penetrated the non-conductive ferrite and MNP samples.

**Figure 2:**
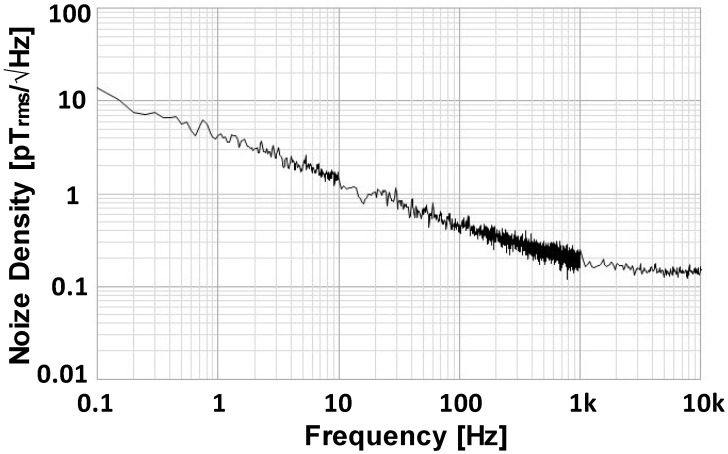
Noise density spectrum of MR sensor (Nivio xMR sensor, TDK Corp.).

An induction-type detection coil is conventionally used to detect a magnetic field generated by a sample. We employed two identical MR sensors (Nivio x MR sensor, TDK Corp., Tokyo, Japan) [17,18,19] based on advanced MR element technology, which represents a significant originality in this work. These sensors were developed for high sensitivity in measuring biomagnetic fields at room temperature in a compact form. Figure 2 shows the noise density spectrum of the sensor, with a noise density of 0.17 pT/Hz^1/2^ (0.12 pT_rms_/Hz^1/2^). The sensitivity of these sensors is comparable to that of superconducting quantum interference devices, enabling measurements of biomagnetic fields such as magnetocardiograms and magnetoencephalograms. Previous studies have demonstrated the sensor’s applicability to low-field MPI systems for detecting MNPs externally excited by low-amplitude magnetic fields (12.4 μT/μ_0_) [19].

The excitation coil was positioned at the center of the two MR sensors, with a distance of 300 mm between the excitation coil and the sensing position of each sensor. These dimensions were chosen considering human body size to facilitate clinical applications of MPI. Measurement samples were placed between the excitation coil and one of the MR sensors (MR sensor 1), as shown in Figure 1. MR sensor 1 measured the magnetic field generated by the samples, positioned *x* = 20 mm away for aluminum, stainless-steel, and ferrite samples and *x* = 5 mm away for MNP samples.

To eliminate the influence of the excitation field, the output signals from the two sensors were canceled by positioning the sensors in opposite directions relative to the excitation field. The positions of the two sensors were adjusted accurately to minimize the total output of the sensors. As a result, only MR sensor 1, which was closer to the sample, detected the magnetic response, while MR sensor 2, positioned farther away, did not. The output signal from the sample was analyzed using a differential amplifier circuit, a lock-in amplifier (NF Corp., LI5645), and an oscilloscope (IWATSU ELECTRIC, Tokyo, Japan, DS-5624A).

The magnetic susceptibility of the MNP samples was measured using a frequency response analyzer (NF Corp., FRA51615), and the complex susceptibility was analyzed [20]. The frequency range for these measurements was *f* = 0.1–10 kHz.

## 4. Results and Discussion

### 4.1. Sensitivity of MR Sensor

**Figure 3:**
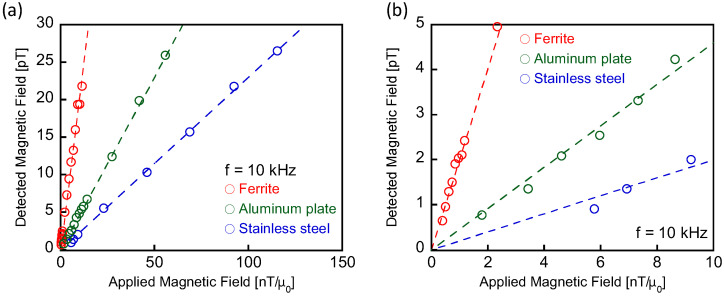
Intensities of magnetic flux densities detected by the MR sensor are a function of the intensity of the applied magnetic field. The magnetic flux was generated in the aluminum, stainless-steel, and ferrite samples. The amplitude and frequency of the applied magnetic field were *H*_ex_ = 0.2–120 nT/μ_0_ and *f* = 10 kHz, respectively. (**b**) provides an enlarged view of the weak-field range depicted in (**a**).

An alternating magnetic field of *H*_ex_ = 0.2–120 nT/μ_0_ at *f* = 10 kHz was applied to the aluminum, stainless-steel, and ferrite samples. The magnetic field generated in the samples was measured using the MR sensor. Figure 3a illustrates the intensities of the magnetic fields detected by the MR sensor. The physical origins of the generated magnetic fields are discussed in the next section. The intensities were proportional to the applied field intensity. As shown in Figure 3b, which provides an enlarged view of the weak applied field range in Figure 3a, we achieve the measurement of the magnetic field from the samples with a high sensitivity below 0.7 pT. This detection limit of magnetic field amplitude by the MR sensor is not affected by the sample position or the distance between the sample and the excitation coil. Notably, a detected signal intensity expressed in units of magnetic field is essential for evaluating detection capabilities in NDT and MPI. Considering the experimental system, which lacked magnetic shielding components, and the noise density of 0.17 pT/Hz^1/2^ at 10 kHz of the MR sensor, this experimentally obtained sensitivity is encouraging for practical applications.

### 4.2. Frequency Dependence of Detected Field Intensity

**Figure 4:**
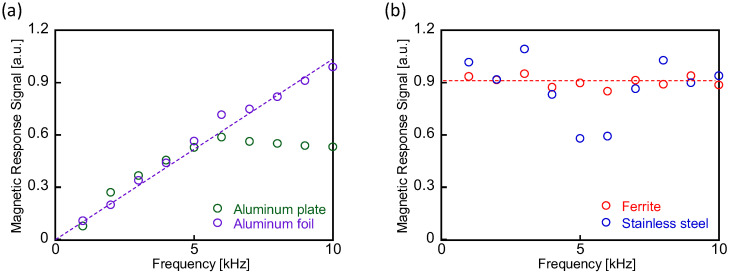
Intensities of magnetic response signals measured from (**a**) aluminum (plate and foil) and (**b**) stainless-steel and ferrite samples as a function of the applied field frequency *f* = 1–10 kHz.

In this section, we introduce the concept of the magnetic response signal, defined as the detected signal intensity normalized by the applied magnetic field intensity, *H*_ex_ (=1.3–26.4 nT/μ_0_). Figure 4 illustrates the frequency dependence of the magnetic response signal intensity. The frequency of the magnetic field applied to the samples ranges from *f* = 1 to 10 kHz. Measurements were conducted on aluminum (plate and foil), stainless-steel, and ferrite samples. The detected intensity of the magnetic response signal for the aluminum foil sample is proportional to the frequency, as shown in Figure 4a. In contrast, the magnetic response signal from the aluminum plate decreases at higher frequencies *(f* > 5 kHz). This difference in frequency dependence between the aluminum foil and plate is caused by their difference in thickness. The skin depth of aluminum at 5 kHz is 1.2 mm. An applied magnetic field penetrates the foil, which has a thickness of 12 μm, and the response signal is not affected by the field frequency. The thickness of the plate, 0.5 mm, is comparable to the skin depth; therefore, the intensity of the applied field weakens during its propagation in the plate. These observations align with the estimation that the physical origin of the generated magnetic field in aluminum is due to eddy currents. For the ferrite sample, the intensity of the magnetic response signal remains constant across the applied frequency range, as shown in Figure 4b. Since ferrite is non-conductive, no eddy currents are generated. The detected signal in this frequency range *f* = 1–10 kHz arises solely from magnetization changes in the material.

Figure 4b also presents the magnetic response signals for the stainless-steel sample. The frequency dependence of the detected signal is not clearly identifiable. This behavior is likely due to a combination of signals originating from both eddy currents and magnetization changes within the material.

### 4.3. Measurement of Magnetic Nanoparticles

**Figure 5:**
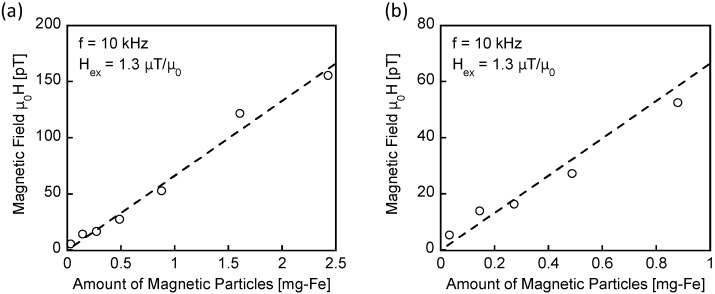
Intensity of the magnetic flux density detected by the MR sensor as a function of the iron content (mg-Fe) in the MNP samples. The magnetic flux was generated by magnetization changes in the MNP samples. Measurements were conducted on 0.1 mL MNP samples containing 0.028–2.43 mg-Fe. The applied magnetic field had an amplitude of *H*_ex_ = 1.3 μT/μ_0_ and *f* = 10 kHz. (**b**) provides an enlarged view of the weak-field range depicted in (**a**).

Figure 5 presents the intensity of the magnetic flux density detected by the MR sensor as a function of the iron content (mg-Fe) in the MNP samples. Measurements were conducted on 0.1 mL MNP samples containing 0.028 to 2.43 mg-Fe. The applied magnetic field has an amplitude of *H*_ex_ = 1.3 μT/μ_0_ and a frequency of *f* = 10 kHz. Figure 5b provides an enlarged view of the weak-field range in Figure 5a. As shown, the detected magnetic flux density is proportional to the iron content in the samples. Notably, a magnetic field of 15 pT is successfully detected from an MNP sample containing 0.14 mg-Fe.

**Figure 6:**
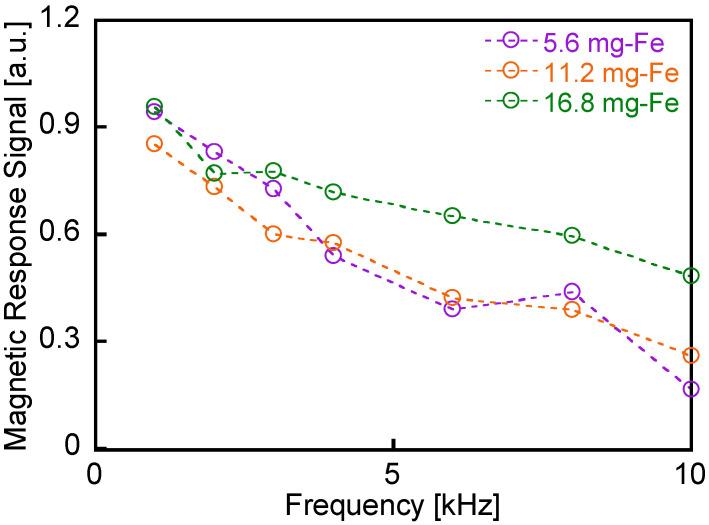
Intensities of magnetic response signals measured from the MNP samples as a function of the applied field frequency *f* = 1–10 kHz. These intensities were calculated from the detected magnetic flux density normalized by the iron content in the samples. Measurements were conducted on 0.1 mL MNP samples containing 5.6, 11.2, and 16.8 mg-Fe.

Figure 6 illustrates the magnetic response signal intensities measured from the MNP samples as a function of the applied magnetic field frequency *f* = 1–10 kHz. These intensities were calculated by normalizing the detected magnetic flux density with respect to the iron content in the samples. Measurements were conducted on 0.1 mL MNP samples containing 5.6, 11.2, and 16.8 mg-Fe. The frequency dependence of the magnetic response signals for the MNP samples differ significantly from those of the aluminum, stainless-steel, and ferrite samples shown in Figure 4. Specifically, the magnetic response signals for the MNP samples decrease with increasing applied field frequency. This behavior aligns with the theory of Brownian relaxation in liquid-phase MNP samples. At higher frequencies, such as 1 kHz, magnetization rotation due to particle rotation is reduced because the particle rotation cannot keep up with the changes in the applied magnetic field [12,13].

**Figure 7:**
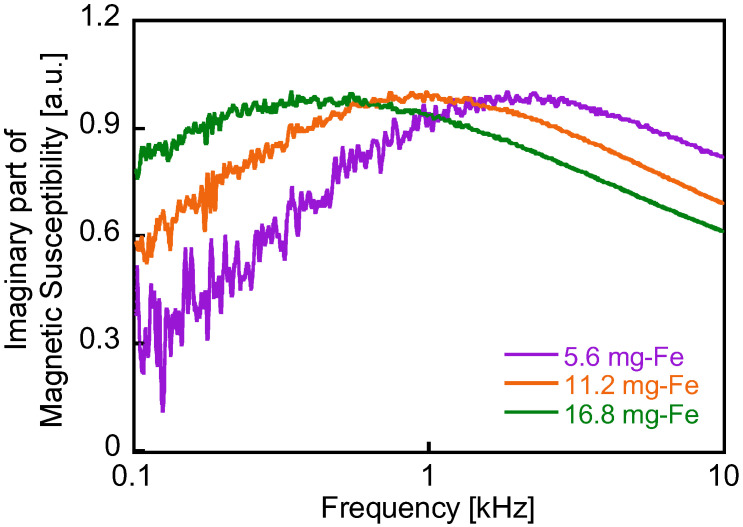
Imaginary parts of the magnetic susceptibility of the MNP samples as a function of the applied field frequency (frequency range of *f* = 0.1–10 kHz). The magnetic susceptibility was normalized by the iron content in the MNP samples. Measurements were conducted on 0.1 mL MNP samples containing 5.6, 11.2, and 16.8 mg-Fe.

The frequency spectra of the magnetic susceptibility of these MNP samples were measured over the frequency range of *f* = 0.1–10 kHz to determine *τ*_B_ of the samples. The measurement system used in this study was capable of measuring AC susceptibility at frequencies below 1 kHz, whereas the detection system shown in Figure 1 had a lower frequency limit of 1 kHz. The magnetic susceptibility was normalized by the iron content in the samples. Figure 7 depicts the imaginary part of the complex susceptibility, *χ*″, which represents the delay in magnetization response to the applied magnetic field. The peak frequencies of *χ*″ vary depending on the iron content in the samples, as shown in Figure 7. This variation is attributed to the differences in MNP density between the samples. At higher densities, magnetization changes are suppressed by magnetic dipole interactions between particles, resulting in longer relaxation times. Consequently, the peak frequency satisfying 2πfτ=1 is lower for samples with higher MNP density [21]. This density-dependent peak shift in the *χ*″ spectrum is clearly observed in Figure 7. From this measurement, *τ*_B_ for the three samples is approximately 1 ms (corresponding to a frequency of 1 kHz), which is consistent with the frequency dependence of the magnetic response signals shown in Figure 6.

## 5. Conclusions

In this study, we successfully detected the magnetic response in samples of aluminum, stainless-steel, ferrite, and magnetic nanoparticles using a measurement system consisting of an excitation coil and highly sensitive magnetoresistive sensors separated by 300 mm. An alternating magnetic field was applied to the samples, and the resulting magnetic response signal was detected using two advanced MR sensors. The system achieved a minimum detectable magnetic field intensity of 0.7 pT. A key direction for future research will be to investigate the minimum detectable sample weight as a function of the sample position between the excitation coil and the sensors. This work paves the way for the development of practical applications in NDT and MPI.

## Data Availability

The data presented in this article are available from the authors on request.
